# Association of a dual-index phenotype and early ICU reassessment with 28-day in-hospital mortality in cardiogenic shock: a single-center observational cohort study

**DOI:** 10.3389/fcvm.2026.1825969

**Published:** 2026-05-14

**Authors:** Rongtai Luo, Zifeng Zeng, Jiajia Li, Huaqing Yao, Weiyong Xu, Xinping Lan

**Affiliations:** Department of Cardiac Intensive Care Unit, Meizhou People’s Hospital, Meizhou, Guangdong, China

**Keywords:** albumin, cardiogenic shock, chloride, hyperchloremia, hypoalbuminemia, in-hospital mortality, intensive care, landmark analysis

## Abstract

**Background:**

Cardiogenic shock (CS) carries high short-term mortality. In this study, we test whether coexisting hypoalbuminemia and hyperchloremia (dual-index phenotype, DIP) and early intensive care unit (ICU) reassessment are associated with 28-day in-hospital mortality.

**Methods:**

In this single-center prospective observational cohort study (January 2021–December 2025), DIP was defined as albumin <35 g/L and chloride >110 mmol/L on paired laboratories within 0–6 h of ICU admission. We used a prespecified 30 h landmark: patients alive and still in the ICU at 30 h formed the analytic cohort, and outcome follow-up began at the landmark. A subset with a second paired assessment at 18–30 h was categorized as non-DIP → non-DIP, non-DIP → DIP, DIP → non-DIP, and DIP → DIP. Sensitivity analyses included baseline-only adjustment, propensity-score overlap weighting, Fine–Gray competing-risk modeling, and exploratory incremental prognostic performance assessment.

**Results:**

Among 982 screened admissions, 444 patients alive and still in the ICU at 30 h comprised the analytic cohort; 74/444 (16.7%) had baseline DIP. The 28-day in-hospital mortality rate was 18.2% (81/444) and was higher in DIP than in non-DIP (37.8% vs. 14.3%). In the 30-h conditional model, baseline DIP remained associated with mortality [adjusted odds ratio (aOR) 2.46, 95% confidence interval (CI) 1.30–4.64; *p* = 0.005]. The results were directionally consistent in a baseline-only model (aOR 2.71, 95% CI 1.45–5.06), a propensity-score overlap-weighted model (OR 2.39, 95% CI 1.27–4.50), and a Fine–Gray competing-risk model [subdistribution hazard ratio (sHR) 2.31, 95% CI 1.35–3.95]. Adding DIP to the clinical landmark model modestly improved discrimination (C-statistic 0.782–0.801). In the reassessment subset (*n* = 96), the mortality rate was 8.3% in non-DIP → non-DIP and 60.0% in DIP → DIP, with wide uncertainty due to small subgroup sizes.

**Conclusions:**

Among CS patients alive and still in the ICU at 30 h, baseline DIP and persistence of DIP by approximately 24 h identified a subgroup at higher risk of 28-day in-hospital death. The association was robust in baseline-only, propensity-weighted, and competing-risk sensitivity analyses, but trajectory findings remained exploratory because of small subgroup sizes. These findings apply to the selected 30 h survivor cohort and should not be used as an ICU-admission prognostic model or extrapolated to early deaths, early ICU transfers, or patients without paired early laboratories without external validation.

## Introduction

Cardiogenic shock (CS) is a life-threatening syndrome of systemic hypoperfusion and end-organ dysfunction driven primarily by cardiac pump failure. Even in the modern era of early reperfusion, advanced critical care, and expanding mechanical circulatory support (MCS), short-term prognosis remains guarded, with many cohorts reporting hospital or 30-day mortality rates that commonly exceed 30% and that can be substantially higher in the sickest patients (1, 2). Beyond mortality, CS frequently culminates in multiorgan failure, prolonged ventilation, acute kidney injury (AKI), and resource-intensive intensive care unit (ICU) stays, creating an urgent need for robust early risk stratification using measures that are objective, rapid, and broadly available.

A persistent barrier to progress is the marked heterogeneity of CS. Etiologies include acute myocardial infarction (AMI), decompensated chronic heart failure, valvular catastrophes, myocarditis, and arrhythmia-mediated collapse, each with distinct hemodynamic profiles and therapeutic priorities (1). Contemporary guidance recommends structured assessment of shock severity using frameworks such as the Society for Cardiovascular Angiography and Interventions (SCAI) shock stages, integrating bedside findings with biochemical and (when available) hemodynamic parameters to support triage, communication, and escalation decisions (3–6). Outcomes remain heterogeneous despite selective use of temporary mechanical circulatory support and modern critical care, underscoring the need for complementary, rapidly available risk markers (7–9).

Routine laboratory indices may offer a practical, scalable layer of phenotyping that complements hemodynamics. Serum chloride is measured repeatedly in most ICU patients and can change rapidly in response to resuscitation practices. Hyperchloremia has biological plausibility for adverse renal effects and dyschloremia has been associated with worse ICU outcomes, although treatment-indication bias remains important (10–18). Serum albumin is another ubiquitous biomarker that reflects physiologic reserve as well as inflammation, capillary leak hemodilution, and organ dysfunction in critical illness. Across acutely ill populations, hypoalbuminemia is consistently associated with worse outcomes, but fluid strategy and albumin administration complicate causal interpretation (19–23).

Importantly, albumin and chloride are usually studied separately, even though their co-occurrence may define a clinically relevant, treatment-sensitive laboratory pattern at the intersection of capillary leak/inflammation, chloride load, and impaired renal handling. DIP should therefore be viewed as an exploratory composite rather than a validated pathobiological syndrome. Early reassessment may capture evolving physiology more effectively than a single baseline value, consistent with dynamic 24 h shock reclassification and renal-risk assessment in CS (24, 25).

We conducted a single-center observational cohort study to evaluate whether the co-occurrence of hypoalbuminemia and hyperchloremia, a dual-index phenotype (DIP), and its early ICU reassessment are associated with 28-day in-hospital mortality in CS, among patients alive and still in the ICU at 30 h, and to describe associations with acute kidney injury and ventilator-free days (VFDs) as exploratory endpoints.

## Methods

### Study design and setting

This was an observational, prospective single-center cohort study conducted at the Meizhou People's Hospital with a mixed medical–cardiac ICU. Consecutive adult ICU admissions with cardiogenic shock were screened between January 2021 and December 2025. Laboratory testing and bedside management were performed according to routine clinical care, and no protocolized interventions were mandated by the study. Data were extracted from the electronic health record (EHR) and institutional clinical information systems and curated into an ICU research registry for analysis.

### Participants, index time, and landmark framework

Eligible participants were adults (≥18 years) admitted to the ICU with cardiogenic shock as the predominant shock etiology. Cardiogenic shock was defined as a clinician-documented diagnosis of CS plus objective evidence of hypotension/vasopressor dependence and hypoperfusion attributable to a primary cardiac cause, consistent with contemporary guideline-style criteria (3–6). Specifically, patients were required to meet both (1) hypotension defined as systolic blood pressure (SBP) <90 mmHg for ≥30 min, mean arterial pressure (MAP) <65 mmHg for ≥30 min, or vasopressor requirement to maintain SBP ≥90 mmHg or MAP ≥65 mmHg; and (2) at least one marker of hypoperfusion, including lactate >2 mmol/L, oliguria (<0.5 mL/kg/h), cool extremities/mottling, or altered mentation not primarily attributable to sedation. Inotrope use alone, without hypotension/vasopressor dependence and hypoperfusion, was not sufficient to define CS.

Primary cardiac causes included acute myocardial infarction, decompensated heart failure, valvular catastrophe, myocarditis, and arrhythmia-mediated collapse. Patients were excluded when a non-cardiac shock process (e.g., distributive/septic, hypovolemic/hemorrhagic, obstructive) was judged to be the dominant driver; overlap (mixed shock) was handled pragmatically by retaining patients when CS was documented as the predominant process despite potential overlap (26).

ICU admission date/time was the index time. Because reassessment required laboratory data from the 18–30 h window, we used a prespecified 30 h landmark to avoid look-ahead (immortal-time) bias: patients alive and still in the ICU at 30 h after ICU admission constituted the analytic cohort, and follow-up for outcomes began at the landmark. Thirty hours was chosen *a priori* because it allowed a clinically relevant ∼24 h reassessment while accommodating routine ICU sampling variation and morning-round workflows within the 18–30 h window (24). Patients who died before 30 h or were no longer in the ICU before 30 h (ICU length of stay <30 h, including transfer out) were excluded. Only the first ICU admission per hospitalization was included. This landmark framework therefore supports conditional inference among patients alive and still in the ICU at 30 h, is not applicable at ICU admission, and should not be interpreted as an admission-time prognostic analysis for all screened CS admissions.

### Laboratory assays and harmonization

Serum chloride and serum albumin were measured in the hospital central laboratory under routine internal quality control and external accreditation standards. In routine practice, chemistry panels were typically obtained on ICU admission and again at approximately 24 h, with additional testing as clinically indicated. Albumin was measured using a dye-binding method (27) and chloride was measured using an ion-selective electrode (ISE) method.

To minimize misclassification from rapid physiologic change and to avoid mixing non-equivalent measurement platforms, DIP was defined using chemistry-panel chloride values and paired chemistry-panel albumin values. If multiple results were present within a window, the first eligible pair was used in the baseline 0–6 h window, whereas the pair closest to 24 h was selected in the 18–30 h reassessment window.

### Dual-index phenotype, phenotype strata, and early reassessment

The primary exposure was the DIP, defined *a priori* as the co-occurrence of hypoalbuminemia and hyperchloremia on an early paired laboratory assessment. Hypoalbuminemia was defined as serum albumin <35 g/L and hyperchloremia as serum chloride >110 mmol/L. A paired albumin–chloride assessment was defined as values obtained from the same blood draw or within a prespecified short interval (≤2 h). Baseline DIP status was determined using the first eligible paired albumin and chloride values obtained within 0–6 h after ICU admission.

For phenotype stratification, patients were additionally classified into a four-level phenotype: neither abnormality, isolated hypoalbuminemia only, isolated hyperchloremia only, or DIP (both abnormalities). For early reassessment, DIP status was reevaluated using a second paired albumin–chloride measurement obtained within 18–30 h after ICU admission. When multiple eligible pairs existed in this window, the pair closest to 24 h was selected. Reassessment patterns were defined as non-DIP → non-DIP (N → N), non-DIP → DIP (N → D), DIP → non-DIP (D → N), and DIP → DIP (D → D).

### Outcome

The primary outcome was 28-day in-hospital mortality, defined as death occurring during hospitalization within 28 days of ICU admission. Outcomes were evaluated from the 30-h landmark through day 28.

For binary endpoints modeled with logistic regression, patients discharged alive before day 28 were classified as not having the in-hospital death event by definition (postdischarge mortality was not captured).

Secondary outcomes (exploratory) included the following: (1) AKI during hospitalization within 28 days, defined using Kidney Disease Improving Global Outcomes (KDIGO) criteria based on serum creatinine and, when available, urine output. Baseline creatinine was the most recent value within 7 days prior to ICU admission, and if unavailable, the first creatinine measured within 6 h of ICU admission was used as baseline; and (2) VFDs today 28, defined as the number of calendar days within the first 28 days after ICU admission during which a patient was alive and free from invasive mechanical ventilation. Patients who died before day 28 were assigned 0 VFDs, and days after extubation were counted only if the patient remained free of invasive ventilation for at least 48 consecutive hours.

### Covariates and data sources

Baseline and landmark covariates were extracted from the ICU EHR, bedside monitoring systems, medication administration records, and the laboratory information system using prespecified definitions. Risk-adjustment variables were selected *a priori* for clinical relevance and interpretability and to limit overfitting given the expected event count.

Demographics included age, sex, and body mass index (BMI). Etiology was categorized as AMI-related vs. non-AMI cardiogenic shock based on treating-team attribution and discharge diagnoses. Documented diabetes mellitus, hypertension, and chronic kidney disease (CKD) were recorded as baseline comorbidities. Chronic kidney disease was defined as a documented history of CKD or an estimated glomerular filtration rate <60 mL/min/1.73 m^2^ for ≥3 months when prior data were available.

Illness severity was summarized using the Sequential Organ Failure Assessment (SOFA) score calculated from the worst values within the first 24 h after ICU admission. Lactate used for adjustment was the first value obtained within 0–6 h after ICU admission (closest to admission if multiple).

Key supportive therapies at the landmark included invasive mechanical ventilation and MCS, defined as intra-aortic balloon pump, extracorporeal membrane oxygenation, percutaneous ventricular assist devices, or other temporary MCS modalities.

Vasopressor/inotrope intensity was quantified by the vasoactive–inotropic score (VIS) at the 30 h landmark calculated as follows: VIS = dopamine (µg/kg/min) + dobutamine (µg/kg/min) + 100 × epinephrine (µg/kg/min) + 100 × norepinephrine (µg/kg/min) + 10 × milrinone (µg/kg/min) + 10,000 × vasopressin (U/min), using the most recent infusion rates prior to the landmark.

### Missing data

Patients without the required paired baseline albumin and chloride measurements in the 0–6 h window were not eligible for baseline DIP analyses. Patients without a second paired measurement in the 18–30 h window were not eligible for reassessment analyses.

Within eligible cohorts, missingness in covariates was addressed using multiple imputation by chained equations under a missing-at-random assumption conditional on observed demographics, comorbidities, severity markers, treatments, exposure status, and outcome indicators (28). Among the 444 eligible patients, missingness for adjusted covariates was low (highest 4.5% for BMI and 3.2% for lactate; all others ≤2.5%). We generated 20 imputed datasets using variable-appropriate imputation models, analyzed each dataset separately, and pooled estimates using Rubin's rules. Outcomes were not imputed. Convergence was assessed by using trace plots and observed vs. imputed distribution checks, which did not suggest instability.

### Statistical analysis

Baseline characteristics were summarized overall by baseline DIP status using mean ± standard deviation (SD) for approximately normally distributed continuous variables, median (interquartile range, IQR) for skewed variables, and counts with percentages for categorical variables. Standardized mean differences (SMDs) were reported to quantify imbalance.

*Primary analysis*: We fit a multivariable logistic regression model in the 30 h landmark cohort and reported adjusted odds ratios (aORs) with 95% confidence intervals (CIs) for determining the association between baseline DIP and 28-day in-hospital mortality. The adjustment set included age, sex, body mass index, diabetes mellitus, AMI etiology, chronic kidney disease, SOFA score, lactate, invasive mechanical ventilation at the landmark, MCS at the landmark, and VIS at the landmark. Because lactate and VIS were right-skewed, both were log-transformed for model stability (natural log). To address potential overadjustment by postbaseline variables, we devised a baseline-only sensitivity model including age, sex, body mass index, diabetes mellitus, AMI etiology, chronic kidney disease, baseline lactate, and baseline creatinine. To examine robustness to baseline imbalance, we additionally fit a propensity-score overlap-weighted logistic model using the same pre-exposure covariates (29).

*Secondary outcomes (AKI and ventilator-free days)*: Secondary outcomes were analyzed as exploratory endpoints and are presented primarily descriptively to avoid overinterpretation, given treatment influence and time-varying severity.

Phenotype stratification compared four baseline phenotype categories with “neither abnormality” as reference and estimated unadjusted odds ratios (ORs) with 95% CIs. Multiplicative interaction for mortality was assessed by including a hypoalbuminemia × hyperchloremia product term in an unadjusted logistic regression model.

Reassessment patterns were analyzed as nominal categories (non-DIP → non-DIP as reference). No ordinal trend test across the four categories was performed because the categories are not inherently ordered. To assess potential selection into the reassessment subset, we descriptively compared mortality and AKI between patients with and without repeat paired testing.

Because discharge alive before day 28 precludes 28-day in-hospital death, we additionally designed a Fine–Gray competing-risk model treating discharge alive as a competing event (30). As a descriptive complementary analysis, we also displayed Kaplan–Meier curves from the 30 h landmark and estimated hazard ratios using Cox proportional hazards regression, recognizing that these methods do not fully account for the competing event of discharge alive. Incremental prognostic performance of DIP beyond the clinical landmark model was explored by using the change in C-statistic, calibration intercept/slope, Brier score, and continuous net reclassification improvement; these analyses were considered exploratory because no external validation sample was available.

Prespecified subgroup analyses were exploratory and evaluated effect modification using interaction terms in the adjusted logistic model. Interaction *p*-values and Benjamini–Hochberg false discovery rate (FDR)-adjusted q-values were reported descriptively and interpreted cautiously. All analyses were conducted in R (version 4.3.2).

## Results

### Participant flow and baseline characteristics

**Figure 1:**
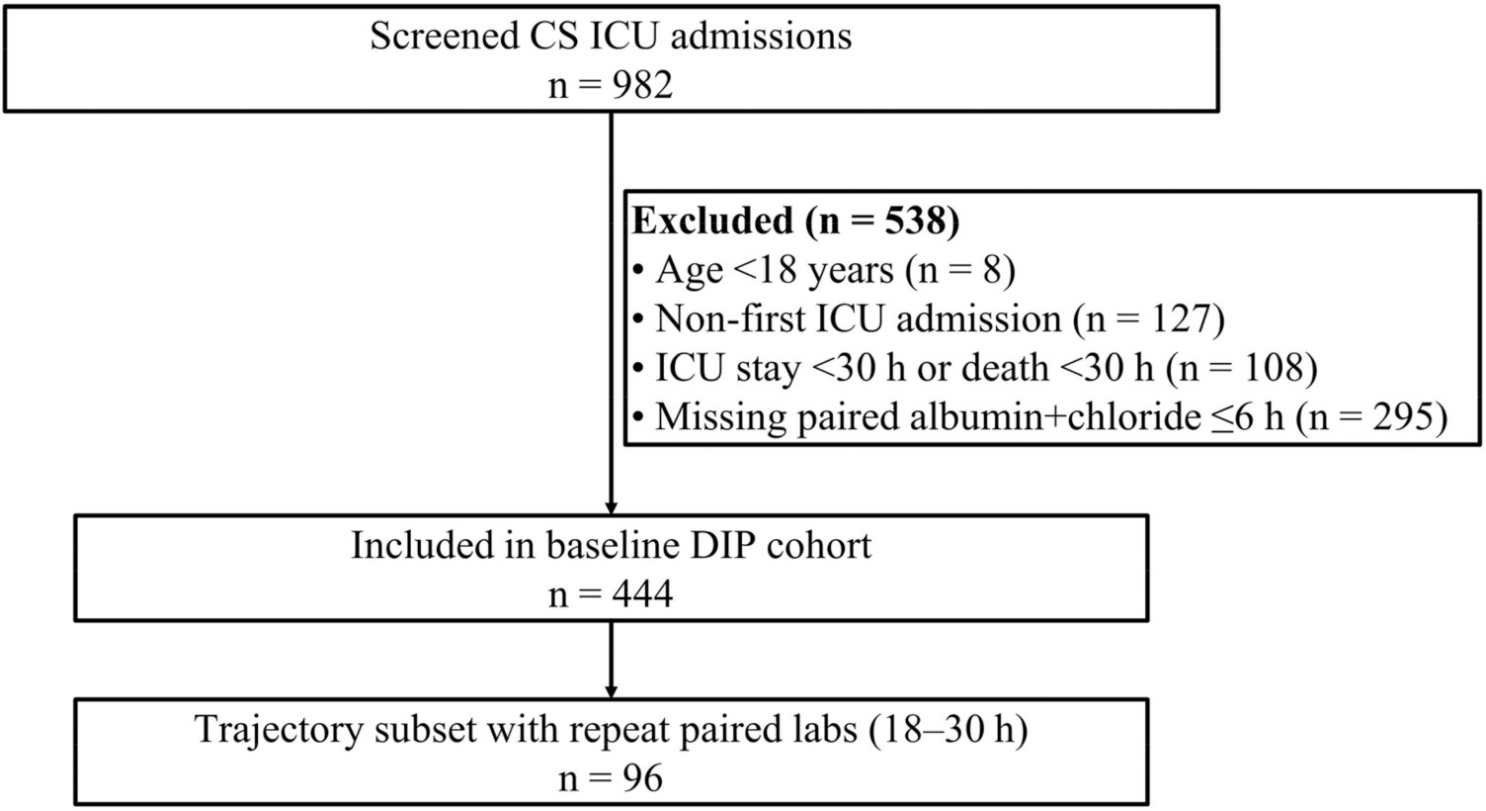
A study flow diagram. A flow diagram of cohort assembly for the baseline DIP analysis cohort (*N* = 444) and the early reassessment subset (*n* = 96).

Following the prespecified eligibility criteria and 30-h landmark framework, 444 patients alive and still in the ICU at 30 h constituted the baseline DIP analytic cohort (Figure 1). Of 982 cardiogenic shock ICU admissions screened, 8 patients aged <18 years, 127 non-first ICU admissions, 108 patients with ICU stay <30 h or death before the landmark, and 295 patients without paired baseline albumin and chloride values within 0–6 h were excluded. Baseline DIP was present in 74/444 (16.7%). Compared with non-DIP patients, DIP patients were older, had higher admission SOFA scores, and more frequently required invasive ventilation at the landmark (Table 1).

**Table 1:** Administration/baseline, first 24 h, and 30 h landmark characteristics among patients alive in the ICU at the 30 h landmark.

Variable	Overall (*N* = 444)	Non-DIP (*n* = 370)	DIP (*n* = 74)	*p*-value	SMD
Admission/baseline (0–6 h after ICU admission					
Age, years	60.8 ± 13.2	60.1 ± 13.2	64.3 ± 12.6	0.010	0.33
Male	310 (69.8%)	260 (70.3%)	50 (67.6%)	0.746	−0.06
Body mass index, kg/m^2^	24.3 ± 3.2	24.5 ± 3.2	23.6 ± 3.0	0.032	−0.27
Acute myocardial infarction etiology	255 (57.4%)	203 (54.9%)	52 (70.3%)	0.020	0.32
Hypertension	245 (55.2%)	202 (54.6%)	43 (58.1%)	0.670	0.07
Diabetes mellitus	131 (29.5%)	99 (26.8%)	32 (43.2%)	0.007	0.35
Chronic kidney disease	76 (17.1%)	58 (15.7%)	18 (24.3%)	0.102	0.22
Lactate within 0–6 h of ICU admission, mmol/L	3 (2–5)	3 (2–4)	4 (3–5)	0.088	0.21
Serum creatinine within 0–6 h of ICU admission, μmol/L	102 (80–129)	101 (80–125)	110 (89–150)	0.012	0.38
Serum albumin within 0–6 h of ICU admission, g/L	36.8 ± 5.2	38.2 ± 4.5	30.0 ± 2.6	<0.001	−2.20
Serum chloride within 0–6 h of ICU admission, mmol/L	107.4 ± 5.0	105.9 ± 4.0	114.8 ± 2.2	<0.001	2.73
First 24 h					
SOFA score (worst first 24 h)	9 (7–11)	9 (7–11)	10 (9–12)	<0.001	0.57
30 h landmark					
Mechanical ventilation at 30 h landmark	283 (63.7%)	224 (60.5%)	59 (79.7%)	0.003	0.42
Mechanical circulatory support at 30 h landmark	175 (39.4%)	141 (38.1%)	34 (45.9%)	0.259	0.16
VIS at 30 h landmark	52 (36–77)	49 (34–74)	70 (48–93)	<0.001	0.48

### Primary outcome: 28-day in-hospital mortality

**Figure 2:**
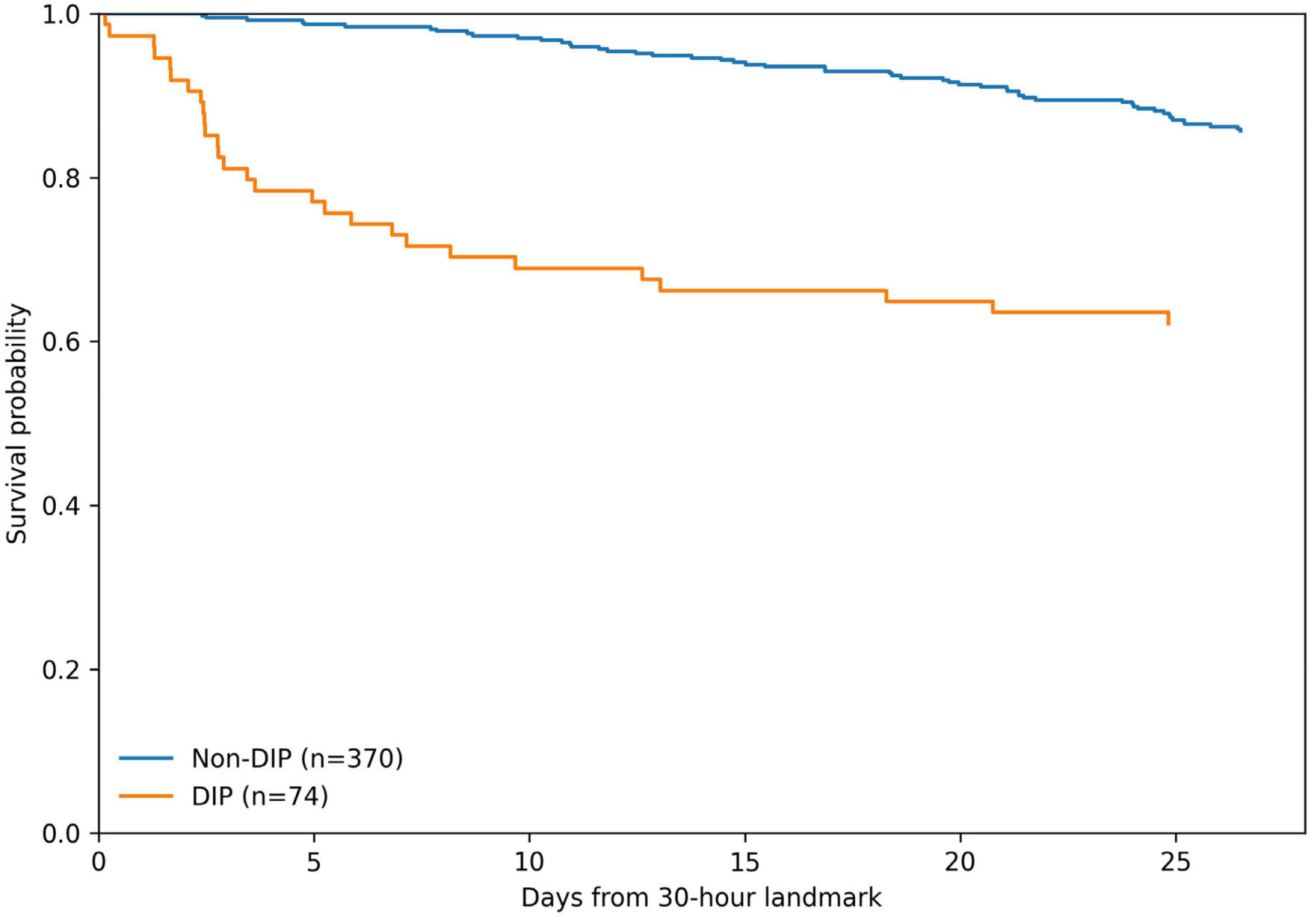
A descriptive Kaplan–Meier survival from the 30 h landmark to day 28 by baseline DIP status. Kaplan–Meier curves compare survival between patients with baseline DIP and those without DIP. Log-rank *p* < 0.001.

Twenty-eight-day in-hospital mortality occurred in 81/444 patients (18.2%) and was higher in the DIP group than in the non-DIP group [37.8% (28/74) vs. 14.3% (53/370)] (Table 2). Baseline DIP was associated with increased odds of death in unadjusted analysis (OR 3.64, 95% CI 2.10–6.33) and remained associated after multivariable risk adjustment in the conditional landmark model including BMI and diabetes (aOR 2.46, 95% CI 1.30–4.64; *p* = 0.005) (Table 3). The results were directionally consistent in a baseline-only model (aOR 2.71, 95% CI 1.45–5.06; *p* = 0.002), a propensity-score overlap-weighted model (weighted OR 2.39, 95% CI 1.27–4.50; *p* = 0.007), and a Fine–Gray competing-risk model treating discharge alive as the competing event [adjusted subdistribution hazard ratio (sHR) 2.31, 95% CI 1.35–3.95; *p* = 0.002]. After overlap weighting, all measured baseline covariates had weighted SMDs <0.10. Adding DIP to the prespecified clinical landmark model modestly improved discrimination [C-statistic 0.782 (0.731–0.833) to 0.801 (0.751–0.851)] with preserved calibration and a continuous net reclassification improvement (NRI) of 0.21 (0.03–0.39) (Supplementary Table S1). Descriptive time-to-event analyses were concordant, with early separation of Kaplan–Meier curves from the 30 h landmark (log-rank *p* < 0.001) (Figure 2) and an adjusted Cox hazard ratio (HR) of 2.34 (95% CI 1.40–3.90; *p* = 0.001) (Table 3).

**Table 2:** Clinical outcomes.

Panel A. Outcomes by baseline DIP status						
Outcome		Non-DIP		DIP	Effect estimate	
28-day in-hospital mortality		53 (14.3%)		28 (37.8%)	RD 23.5% (11.9%–35.1%); OR 3.64 (2.10–6.33)	
Acute kidney injury (KDIGO any stage)		147 (39.7%)		48 (64.9%)	RD 25.1% (13.2%–37.1%); OR 2.80 (1.66–4.71)	
Ventilator-free days within 28 days, days		24 (15–28)		13 (0–24)	Descriptive [median (IQR)]	
Panel B. Outcomes by baseline albumin/chloride phenotype (reference: Neither abnormality)						
Phenotype	n	Mortality	Mortality OR vs. neither	AKI	AKI OR vs. neither	VFD, median (IQR)
Neither	246	30 (12.2%)	1.00 (ref)	90 (36.6%)	1.00 (ref)	24 (17–28)
Hypoalbuminemia_only	78	14 (17.9%)	1.57 (0.79–3.15)	34 (43.6%)	1.34 (0.80–2.25)	22 (14–28)
Hyperchloremia_only	46	9 (19.6%)	1.75 (0.77–3.99)	23 (50.0%)	1.73 (0.92–3.27)	21 (10–28)
DIP	74	28 (37.8%)	4.38 (2.39–8.03)	48 (64.9%)	3.20 (1.86–5.51)	13 (0–24)

RD, risk difference; VFD, ventilator-free days.

**Table 3:** Primary and sensitivity analyses for the association between baseline DIP and 28-day in-hospital mortality in the 30 h landmark cohort.

Model	Measure	Effect (DIP vs. non-DIP)	*p*-value	*N*	Events
Multivariable logistic regression (landmark model)	Adjusted OR	2.46 (1.30–4.64)	0.005	444	81
Baseline-only logistic regression sensitivity	Adjusted OR	2.71 (1.45–5.06)	0.002	444	81
Propensity-score overlap-weighted logistic regression sensitivity	Weighted OR	2.39 (1.27–4.50)	0.007	444	81
Fine–Gray competing-risk regression	Adjusted sHR	2.31 (1.35–3.95)	0.002	444	81
Cox proportional hazards regression (descriptive)	Adjusted HR	2.34 (1.40–3.90)	0.001	444	81

### Secondary outcomes: acute kidney injury and ventilator-free days

Acute kidney injury occurred in 195/444 patients (43.9%) and was more common with DIP than without [64.9% (48/74) vs. 39.7% (147/370)] (Table 2). Ventilator-free days to day 28 were lower in DIP patients [median 13 (0–24)] than in non-DIP patients [median 24 (15–28)] (Table 2). Because these endpoints are influenced by evolving severity and management, we present them as exploratory descriptive associations.

### Phenotype stratification and interaction

In phenotype stratification (Table 2), the mortality rate was 12.2% in patients with neither abnormality, 17.9% with isolated hypoalbuminemia, 19.6% with isolated hyperchloremia, and 37.8% when both abnormalities co-occurred (DIP). Relative to the neither-abnormality reference, DIP had the highest unadjusted odds of death (OR 4.38, 95% CI 2.39–8.03), while isolated abnormalities were associated with more modest elevations. The unadjusted multiplicative interaction term was not statistically significant (*p* = 0.414). This did not exclude potential biological interaction between albumin and chloride; rather, we did not detect statistical interaction on the multiplicative scale and additive interaction was not evaluated.

### Early DIP reassessment (0–6 to 18–30 h)

Among the 96 patients with paired baseline and 18–30-h measurements, reassessment patterns were N → N in 60 (62.5%), N → D in 14 (14.6%), D → N in 12 (12.5%), and D → D in 10 (10.4%) (Table 4). Baseline characteristics across the four trajectory groups are given in Supplementary Table S2 and suggest less favorable baseline profiles in patients with persistent DIP. The mortality rate was 8.3% (5/60) in N → N, 28.6% (4/14) in N → D, 25.0% (3/12) in D → N, and 60.0% (6/10) in D → D. Compared with N → N, persistent DIP (D → D) had the highest crude odds of death (OR 16.50, 95% CI 3.46–78.65, *p* < 0.001), and new-onset DIP (N → D) was also associated with higher mortality (OR 4.40, 95% CI 1.00–19.28; *p* = 0.049). Secondary outcomes were concordant: AKI occurred in 20/60 (33.3%) with N → N, 8/14 (57.1%) with N → D, 5/12 (41.7%) with D → N, and 8/10 (80.0%) with D → D, alongside the lowest ventilator-free days in the persistent-DIP group (Table 4). To assess potential selection, overall mortality and AKI in this reassessment subset were similar to patients without repeat paired testing [18/96 (18.8%) vs. 63/348 (18.1%) for mortality; 41/96 (42.7%) vs. 154/348 (44.3%) for AKI], suggesting limited outcome-related selection for the trajectory analysis.

**Table 4:** Exploratory early DIP reassessment patterns and outcomes.

Trajectory	*n*	28-day in-hospital mortality	OR vs. N → N	*p*	AKI	VFD, median (IQR)
N → N	60	5 (8.3%)	1.00 (ref)		20 (33.3%)	28 (21–28)
N → D	14	4 (28.6%)	4.40 (1.00–19.28)	0.049	8 (57.1%)	20 (2–25)
D → N	12	3 (25.0%)	3.67 (0.74–18.08)	0.110	5 (41.7%)	24 (8–26)
D → D	10	6 (60.0%)	16.50 (3.46–78.65)	<0.001	8 (80.0%)	0 (0–10)

### Exploratory subgroup analyses

**Figure 3:**
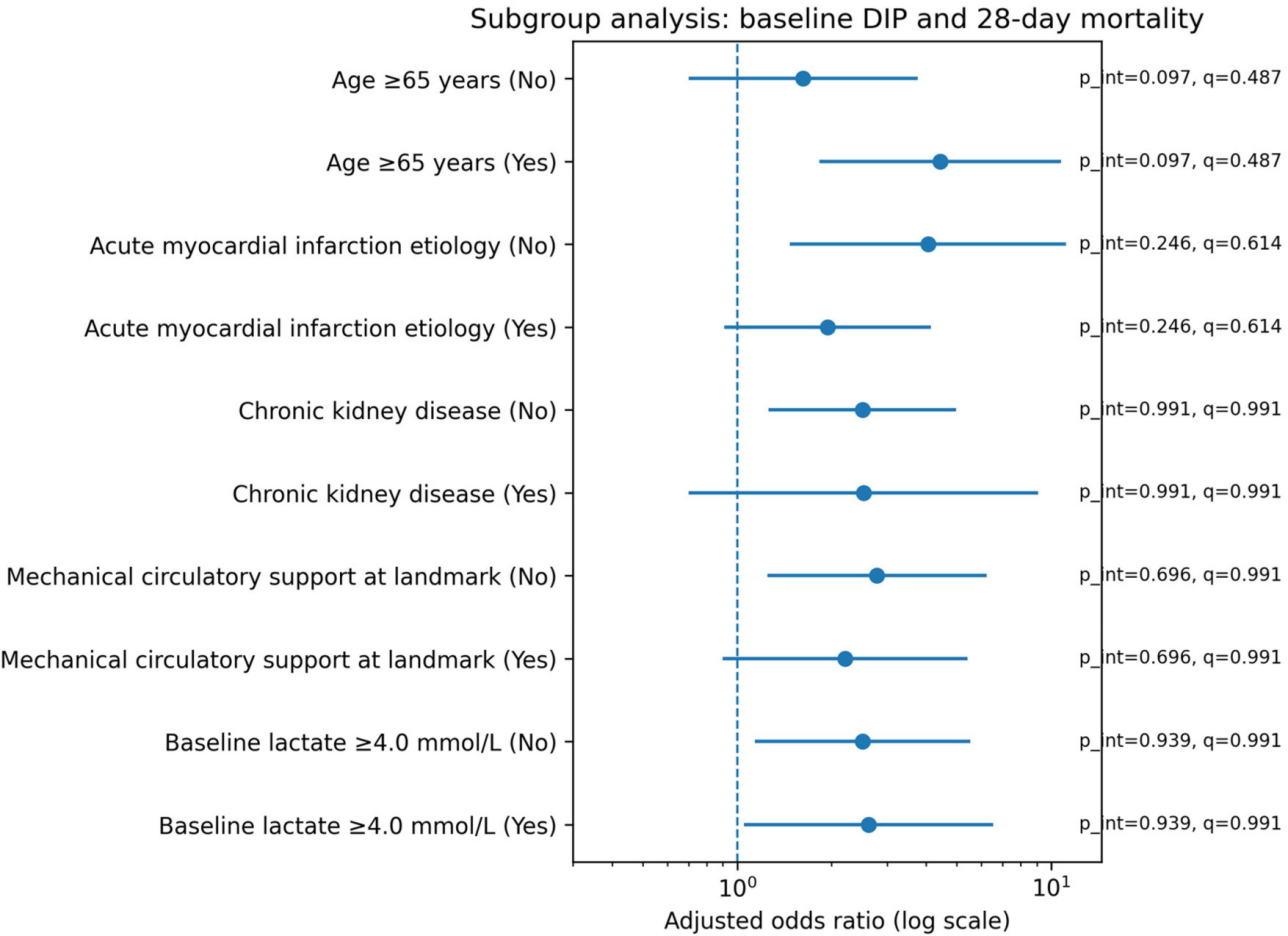
An exploratory subgroup analysis of baseline DIP and 28-day in-hospital mortality. A forest plot of adjusted ORs for baseline DIP versus non-DIP within prespecified subgroups. ORs and 95% CIs are derived from models with DIP × subgroup interaction terms; p_int values are interaction *p*-values and *q*-values are Benjamini–Hochberg FDR-adjusted across interactions.

In exploratory subgroup analyses (Figure 3), adjusted associations were directionally consistent, and no interaction remained significant after FDR adjustment for interaction testing (all *q* ≥ 0.487). Numerically larger point estimates were observed in older patients, AMI-related CS, and higher-lactate strata, but formal interaction testing did not provide strong evidence of effect modification (all interaction *p*-values > 0.05). These subgroup findings are therefore descriptive and should be interpreted cautiously given limited power for interaction testing.

## Discussion

In this single-center observational cohort of adults with cardiogenic shock, a simple laboratory-based DIP, concurrent hypoalbuminemia and hyperchloremia within 0–6 h, identified a higher-risk subgroup among patients alive and still in the ICU at the prespecified 30 h landmark. Among 444 eligible patients, DIP was present in 16.7% and the 28-day in-hospital mortality rate was 18.2%. Mortality was higher with DIP than without, and the association persisted in the conditional model, the baseline-only sensitivity model, the propensity-score overlap-weighted analysis, and the competing-risk analysis. Adding DIP to the clinical landmark model also produced a modest improvement in discrimination. In the reassessment subset, the persistence of DIP by approximately 24 h was associated with particularly high mortality, but these small-sample trajectory findings should be viewed as exploratory.

These associations are biologically plausible but should be interpreted as associated rather than causal. Albumin integrates physiologic reserve and acute inflammatory–capillary leak states, and low albumin is consistently associated with worse outcomes across critical illness (19–22). In CS, hypoalbuminemia may also reflect hemodilution from resuscitation, hepatic congestion or impaired synthesis, renal losses, and endothelial dysfunction (19–23). Chloride abnormalities often track both underlying renal vulnerability and treatment exposure, including chloride-rich fluid strategy and other resuscitation practices (10–18). Experimental and human physiologic studies support that hyperchloremia and saline loading can reduce renal blood flow and cortical perfusion, plausibly via chloride-mediated effects on renal vascular tone and tubuloglomerular feedback (10–12). At the same time, trials comparing balanced crystalloids with saline have reported mixed results overall, suggesting that any average benefit is modest and may depend on patient mix, exposure intensity, and outcomes selected (13–16). Observational data also associate dyschloremia with adverse outcomes, although confounding remains important (17, 18). Accordingly, DIP likely captures a convergence of physiologic vulnerability and early management context. We did not detect evidence of multiplicative interaction between hypoalbuminemia and hyperchloremia, but non-significance does not establish absence of interaction, and additive interaction was not assessed. Because fluid composition, fluid balance, diuretic exposure, albumin administration, and granular hemodynamics were not fully captured, residual confounding by treatment indication remains likely. DIP is therefore better regarded as an exploratory, treatment-sensitive laboratory pattern than as a validated stand-alone syndrome.

Our findings support the concept that routine laboratory patterns can complement structured shock staging and, when available, hemodynamic assessment. The SCAI staging framework and more recent consensus guidance emphasize early recognition, multidisciplinary evaluation, and escalation of vasoactive therapy and temporary mechanical circulatory support when needed (3–6). Randomized data for some commonly used technologies remain mixed, highlighting the importance of better phenotyping and targeted trials (7–9). Prior work links dyschloremia and chloride exposure to kidney-related outcomes and mortality (17, 18), and hypoalbuminemia remains a robust prognostic marker in acute and critical illness (20). Our early reassessment findings align with evidence that short-interval reassessment can improve risk stratification in shock (24) and with data that AKI is common and prognostically important in CS (25). DIP also provided a modest improvement in discrimination, calibration, and reclassification beyond the prespecified clinical landmark model, although these performance estimates were only internally derived and SCAI stage was not available with sufficient completeness for a robust head-to-head comparison.

Clinically, DIP should be interpreted as an adjunctive prognostic signal that complements guideline-consistent CS evaluation (etiology determination, severity staging, and hemodynamic assessment when available) (3–6, 31). Because albumin and chloride are routinely available, baseline DIP and reassessment at approximately 24 h should be externally validated and reanalyzed with baseline-only covariate adjustment before any automated implementation or trial-enrichment use is considered. The subgroup analyses showed numerically larger point estimates in some strata, particularly older patients and AMI-related CS, but no interaction survived FDR adjustment; these findings are therefore descriptive and may reflect greater baseline vulnerability rather than true effect modification. Patients with new-onset or persistent DIP may require a deliberate review of acid–base status, kidney-protective practices, and overall resuscitation strategy, including a consideration of chloride load when otherwise clinically appropriate. However, causality cannot be inferred from this observational analysis.

Several limitations in this study should be acknowledged. First, this was a single-center study, and local practice patterns may limit transportability. Second, because this was a 30 h landmark analysis, the findings apply only to those patients who survived and remained in the ICU until the landmark and may not generalize to early deaths or early ICU transfers. Accordingly, the model is not applicable at ICU admission and should be interpreted as conditional on 30 h survival and ICU retention. Of the 538 excluded admissions, 108 had ICU length of stay <30 h or died before the landmark and 295 lacked paired early laboratories; the analytic sample should therefore be interpreted as a highly selected conditional survivor cohort rather than a consecutive admission-time CS cohort. Third, eligibility required paired baseline albumin and chloride testing within 6 h. Many screened admissions lacked these measurements, raising the possibility of additional selection bias related to clinician concern or workflow. Fourth, residual confounding is likely because DIP may reflect unmeasured factors such as total fluid volume and composition, diuretic exposure, albumin administration, and granular hemodynamics, although the revised baseline-only, propensity-weighted, and competing-risk sensitivity analyses were directionally consistent. Finally, reassessment analyses were restricted to a small subset (*n* = 96), producing wide confidence intervals for some comparisons. In addition, the main adjusted model incorporated postbaseline variables (worst first-24 h SOFA and 30 h support intensity), and therefore, it should be interpreted as a conditional landmark model rather than a pure admission-time prognostic model.

## Conclusion

Early concurrent hypoalbuminemia and hyperchloremia identified a higher-risk subgroup among cardiogenic shock patients alive and still in the ICU at 30 h and showed modest incremental prognostic value beyond the clinical landmark model. Early reassessment suggested that persistence DIP by ∼24 h may further stratify prognosis, but these trajectory findings are exploratory. Because DIP is treatment-sensitive and this analysis was conditioned on 30 h survival and ICU retention, external validation is required before clinical implementation.

## Data Availability

The raw data supporting the conclusions of this article will be made available by the authors without undue reservation.

## Supplementary material

Supplementary Table S1 Exploratory incremental prognostic performance of DIP beyond the clinical landmark model.

Supplementary Table S2 Baseline characteristics of the four early DIP trajectory phenotypes.
