# Supplementary material for: Association of a dual-index phenotype and early ICU reassessment with 28-day in-hospital mortality in cardiogenic shock: a single-center observational cohort study
Source: Front Cardiovasc Med. 2026 May 14;13:1825969. doi: 10.3389/fcvm.2026.1825969 (PMC13216494; doi:10.3389/fcvm.2026.1825969)
Supplement: Supplementary Table S1 — Exploratory incremental prognostic performance of DIP beyond the clinical landmark model. [file Datasheet1.docx]

**Supplementary Table S1. Exploratory incremental prognostic performance of DIP beyond the clinical landmark model**

| **Model** | **C-statistic (95% CI)** | **Calibration intercept** | **Calibration slope** | **Brier score** | **Continuous NRI vs clinical model** | **LR test p-value** |
| --- | --- | --- | --- | --- | --- | --- |
| Clinical landmark model without DIP | 0.782 [0.731, 0.833] | -0.04 | 0.98 | 0.127 | Reference | Reference |
| Clinical landmark model + DIP | 0.801 [0.751, 0.851] | -0.02 | 0.96 | 0.123 | 0.21 [0.03, 0.39] | 0.011 |

**Supplementary Table S2. Baseline characteristics of the four early DIP trajectory phenotypes**

| **Variable** | **N→N (n=60)** | **N→D (n=14)** | **D→N (n=12)** | **D→D (n=10)** | **p-value** |
| --- | --- | --- | --- | --- | --- |
| Age, years | 57.9 ± 12.4 | 63.1 ± 11.9 | 61.8 ± 12.3 | 68.4 ± 10.8 | 0.041 |
| Male | 44 (73.3%) | 8 (57.1%) | 7 (58.3%) | 6 (60.0%) | 0.401 |
| Body mass index, kg/m^2^ | 24.7 ± 3.0 | 23.8 ± 3.1 | 23.5 ± 2.8 | 22.7 ± 2.6 | 0.089 |
| Acute myocardial infarction etiology | 31 (51.7%) | 9 (64.3%) | 8 (66.7%) | 8 (80.0%) | 0.247 |
| Diabetes mellitus | 14 (23.3%) | 5 (35.7%) | 4 (33.3%) | 6 (60.0%) | 0.048 |
| Chronic kidney disease | 8 (13.3%) | 3 (21.4%) | 2 (16.7%) | 4 (40.0%) | 0.118 |
| SOFA score (worst first 24 h) | 8 [6–10] | 9 [7–11] | 10 [8–11] | 11 [10–13] | 0.006 |
| Lactate within 0–6 h of ICU admission, mmol/L | 2.8 [1.9–4.0] | 3.5 [2.4–5.1] | 3.8 [2.7–4.8] | 4.4 [3.4–5.6] | 0.021 |
| Serum creatinine within 0–6 h of ICU admission, μmol/L | 98 [77–120] | 104 [84–131] | 112 [90–142] | 126 [96–166] | 0.032 |
| Serum albumin within 0–6 h of ICU admission, g/L | 37.9 ± 4.0 | 36.2 ± 4.4 | 31.1 ± 2.7 | 29.1 ± 2.3 | <0.001 |
| Serum chloride within 0–6 h of ICU admission, mmol/L | 105.3 ± 3.7 | 106.7 ± 4.1 | 113.9 ± 2.2 | 115.2 ± 2.0 | <0.001 |
